# Destruction of Jugular Vein and Carotid Artery Following Injection of Barium Chloride

**Published:** 1899-08

**Authors:** George L. Warner

**Affiliations:** New York City


Destruction of Jugular Vein and Carotid Artery Following the Intravenous Injection of Barium Chloride. Dr. George L. Warner, of New York City, reports the following interesting case:

*Subject.* Gray gelding, used by a retail butcher.

*History.* Had been treated previously for flatulency. At time of my examination all evidence of this had disappeared; bowels moving, though scantily; temperature, 102° F. At the right inferior cervical region the skin along the jugular groove had a dried appearance, and over an area 14 inches long by 1½ inches wide looked as if the hair was clipped off. The submaxillary artery was soft, but pulse not perceptible.

*Diagnosis.* Destruction of jugular vein and carotid artery with surrounding tissue as shown by the destroyed skin. I learned from the attending veterinarian at time of flatulency that he had given three injections of barium chloride of ten-grain doses, two on the left side, which, undoubtedly, had been given intravenously, as they left no visible effect, while the one on the right side, probably due to the animal's struggles, had entered subcutaneously. In about three days the separation of the necrosed parts occurred, leaving a large healthy surface, the vein and artery appearing as two dark cords collapsed, and taking with it about eighteen inches of the mastoidio-humeral muscle. In about ten days the case was ready for work, though dressing of the wound was continued for a time every second day. For several days arteritis, to a certain extent, marked by tumefaction and extreme sensibility of the parts, persisted. The head was carried to the right side for a time, but this gradually returned to a normal position.

Having used barium chloride considerably, with no ulterior effects, I was more than surprised to learn of such destructive changes following the administration of ten-grain doses, all of which suggest the more conservative uses of these drugs as recommended.

